# A Three-Pulse Release Tablet for Amoxicillin: Preparation, Pharmacokinetic Study and Physiologically Based Pharmacokinetic Modeling

**DOI:** 10.1371/journal.pone.0160260

**Published:** 2016-08-01

**Authors:** Jin Li, Hongyu Chai, Yang Li, Xuyu Chai, Yan Zhao, Yunfan Zhao, Tao Tao, Xiaoqiang Xiang

**Affiliations:** 1 National Pharmaceutical Engineering Research Center, China State Institute of Pharmaceutical Industry, Shanghai, China; 2 Camelot Academy, Durham, the United States of America; 3 Department of Clinical Pharmacy, School of Pharmacy, Fudan University, Shanghai, China; National University of Singapore, SINGAPORE

## Abstract

**Background:**

Amoxicillin is a commonly used antibiotic which has a short half-life in human. The frequent administration of amoxicillin is often required to keep the plasma drug level in an effective range. The short dosing interval of amoxicillin could also cause some side effects and drug resistance, and impair its therapeutic efficacy and patients’ compliance. Therefore, a three-pulse release tablet of amoxicillin is desired to generate sustained release *in vivo*, and thus to avoid the above mentioned disadvantages.

**Methods:**

The pulsatile release tablet consists of three pulsatile components: one immediate-release granule and two delayed release pellets, all containing amoxicillin. The preparation of a pulsatile release tablet of amoxicillin mainly includes wet granulation craft, extrusion/spheronization craft, pellet coating craft, mixing craft, tablet compression craft and film coating craft. Box–Behnken design, Scanning Electron Microscope and *in vitro* drug release test were used to help the optimization of formulations. A crossover pharmacokinetic study was performed to compare the pharmacokinetic profile of our in-house pulsatile tablet with that of commercial immediate release tablet. The pharmacokinetic profile of this pulse formulation was simulated by physiologically based pharmacokinetic (PBPK) model with the help of Simcyp^®^.

**Results and Discussion:**

Single factor experiments identify four important factors of the formulation, namely, coating weight of Eudragit L30 D-55 (X_1_), coating weight of AQOAT AS-HF (X_2_), the extrusion screen aperture (X_3_) and compression forces (X_4_). The interrelations of the four factors were uncovered by a Box–Behnken design to help to determine the optimal formulation. The immediate-release granule, two delayed release pellets, together with other excipients, namely, Avicel PH 102, colloidal silicon dioxide, polyplasdone and magnesium stearate were mixed, and compressed into tablets, which was subsequently coated with Opadry^®^ film to produce pulsatile tablet of amoxicillin. *In vitro* release study firstly indicated a three-pulse release profile of the tablet. Later the pulse tablet was found to generate the sustained release of amoxicillin in beagle dogs. Furthermore, the Simcyp^®^ software was used to simulate the *in vivo* concentration time curve model of the three-pulse release tablet for amoxicillin in both human and beagle dog. The prediction by PBPK model nicely fitted the observation in human and beagle dog.

**Conclusions:**

This study has demonstrated the interrelation of factors affecting the pulsatile formulation of amoxicillin using a Box–Behnken design. The three-pulse release tablets of amoxicillin were proven to generate pulsatile release *in vitro* and sustained release *in vivo*. This formulation was also found to extend the effective plasma concentration in human compared to the tablet of immediate release based on the simulation data by PBPK modeling. This study provides an example of using PBPK to guide the development of pulsatile dosage forms.

## Introduction

Amoxicillin is one of the most common semi synthetic antibiotics with broad antibacterial spectrum. The half-life of amoxicillin is about 60 minutes in human [1]. Therefore, the routine regimen using conventional dosage forms requires three or four times of drug administration a day to keep the plasma drug levels in an effective range [2]. Frequent administration might cause side effects, generate drug resistance, reduce the effectiveness and result in the poor compliance. Hence, a once daily oral dosage is desired to reduce the frequency of the drug administration. One approach of preparing a once daily oral dosage form is to make the pulsatile release tablet of amoxicillin.

Different from a single pulse formulation, a multiple pulse formulation delivers more than one pulse concomitantly. Each pulse corresponds to a specific lag time [3]. The first pulse is designed to dissolve in the stomach, leading to an immediate release. The subsequent pulses are usually designed to provide a lag time [4, 5]. A pulsatile release tablet of amoxicillin rather than common tablet can better control amoxicillin release, and antibiotics contact with bacteria [6].

A pulse release tablet of amoxicillin was first developed by the MinddleBrook Company in the United States. The drug was called MOXATG^®^ and approved by FDA in 2008. The extended release tablet consists of three pulsatile components: one immediate-release pulse and two types of delayed release pellet. The pulsatile release is controlled by the pH-sensitive coating. The pH-sensitive coating is dissolved gradually with the increase of pH along the gastrointestinal tract. A gradual increase in pH is observed through the human small intestine: pH 5 ~ 6 at the duodenum, pH 6 ~ 6.6 at the jejunum, and pH 6.5 ~ 8 at the ileum [7]. Cellulose derivatives and copolymers of methacrylic acid are traditionally utilized as enteric coating agents [8]. Hypromellose acetate succinate (HPMCAS) is a cellulose derivative bearing succinate groups and hydrophilic enteric coating material [9, 10]. HPMCAS is insoluble in acidic media and dissolves step-wise at pH values greater than 6.0 [11]. Methacrylic acid copolymer (Eudragit L30 D-55) is also insoluble in acidic media and dissolves step-wise at pH values greater than 5.5 [12].

The physiologically based pharmacokinetic model (PBPK) is a kind of *in silico* technique to predict the pharmacokinetic profile of a certain molecule *in vivo*, aiming to make the best use of anatomical and physiological characteristics of humans or animals, the physic-chemical parameters of drug molecules and also the formulation properties. The PBPK has demonstrated its big potential in many aspects of pharmacokinetic research, such as drug-drug interaction, pharmacokinetics in pediatrics and pregnant women, and prediction of first-in-human dose. PBPK has also been found to very useful for the formulation development. For example, Simcyp^®^, the commercial PBPK software, has been used to estimate the bioperformance of oral solid dosage forms [13]. Similarly, a PBPK model built in the software of STELLA has ever been employed to predict the pharmacokinetic profiling of diclofenac in the multiple-unit enteric coated dosage form [14].

The goal of this investigation was to prepare an oral pulsatile release tablet of amoxicillin as a once daily oral dosage form. Moreover, the investigation was to study the internal cause among the formulated factors of the pulsatile release tablet by the single factor experimental design and the Box–Behnken design. The pharmacokinetic behavior of this formulation was evaluated by both the animal model and PBPK technique.

## Materials and Methods

### Chemicals and materials

**Fig 1:**
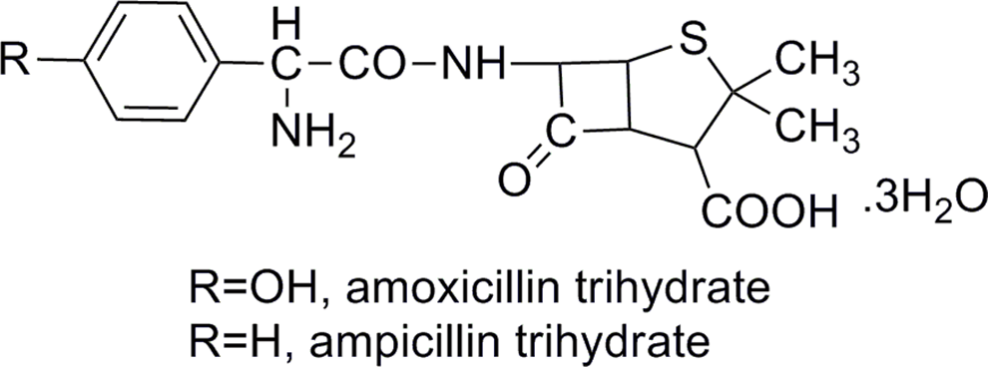
Chemical structure of (a) amoxicillin trihydrate, (b) ampicillin trihydrate is internal standard.

Amoxicillin tryhydrate was supplied by the United Laboratories Co., Ltd (ZhuHai, China). The working standard of amoxicillin trihydrate (85.8% purity) and ampicillin trihydrate (Internal standard, IS) (Fig 1) were obtained from National institutes for food and drug control (Beijing, China). Eudragit L30 D-55 was received as a gift sample from Rohm Pharmaceutical Co., Ltd (Shanghai, China). HPMCAS was given by ShinEtsu Chemical Co., Ltd. (Tokyo, Japan). Microcrystalline cellulose (MCC, Avicel^®^ PH 101, PH 102, PH 301) were from Asahi Kasei Corporation in Japan. Triethyl citrate (TEC) was given by AnHui BBCA Pharmaceutical Co., Ltd (AnHui, China). Polyplasdone (PVPP, Ashalnd, USA) was given from Industrial Polychemical Service Corporation (NewJersey, USA). Sodium hydroxide (GR grade), sodium lauryl sulfate (GR grade), sodium dihydrogen phosphate (GR grade) and phosphoric acid (GR grade) and glacial acetic acid (GR grade) were purchased from Sinopharm Chemical Reagent Co., Ltd (Shanghai, China). Methanol (HPLC grade) and acetonitrile (HPLC grade) were purchased from Merck Pvt. Ltd. (Shanghai, China). Polyoxyl 35 castor oil was purchased from BASF (Germany). Hydroxypropyl methyl cellulose (HPMC E5) and Opadry® were purchased from Colorcon (Shanghai, China).

### Preparation methods and optimization

**Fig 2:**
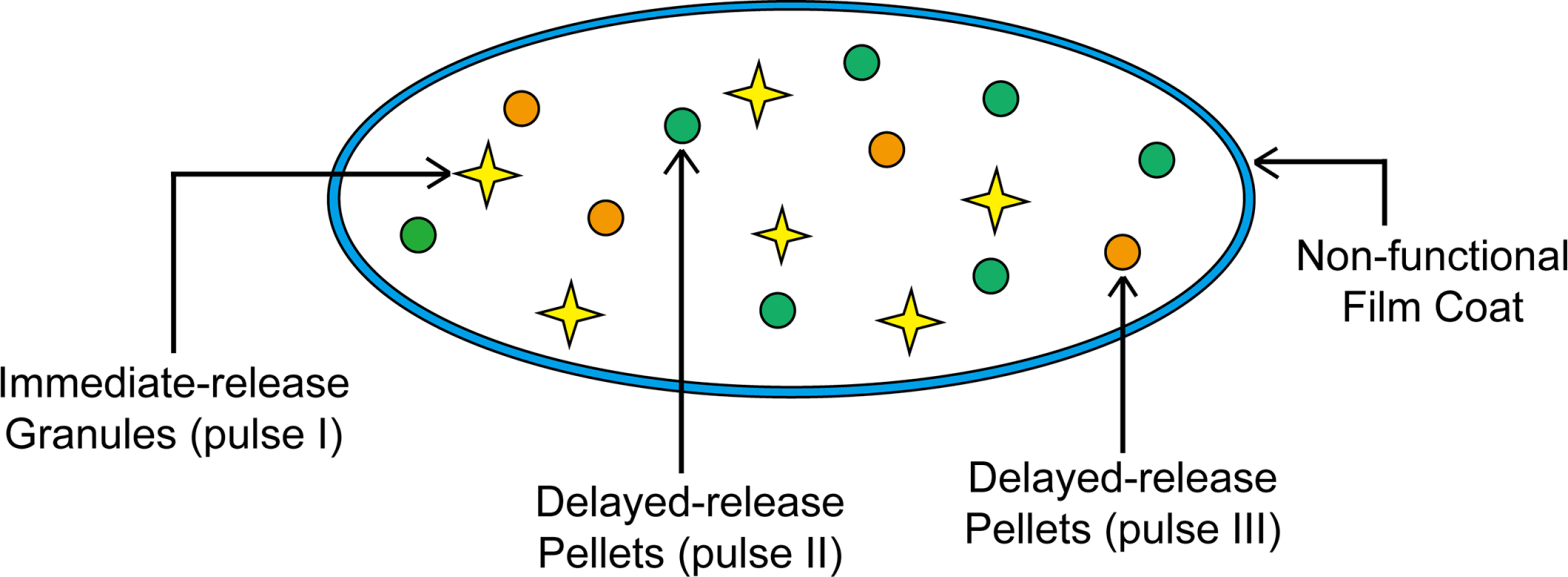
Schematic diagram of amoxicillin pulsatile release tablet.

The amoxicillin tablet was designed to consist of three kinds of pulses, including the pulsatile immediate-release (the pulse Ⅰ) and two pulsatile delayed-releases (the pulse Ⅱ and Ⅲ) (Fig 2).

#### PulseⅠ

The pulse Ⅰ was prepared by the wet granulation craft. The pulse Ⅰ was made of amoxicillin, excipient Avicel MCC, PVPP and the binder HPMC E5 solution. Amoxicillin, Avicel MCC, and PVPP which were firstly mixed for 3 min at 300 rpm by the wet-mixing granulator, and were subsequently mixed with HPMC E5 solution as the binder through a 40-mesh sieve. Finally, the wet granule was dried in an oven at 50°C for 3 h.

#### Preparation of Drug Containing Cores

The extrusion/spheronization process is a well-accepted method of producing pellets [15]. Amoxicillin pellets were prepared by the extrusion/spheronization machine (Mini250, Shenzhen XinYite science and technology co. Ltd, Shenzhen, China). Amoxicillin and Avicel PH 301 were pre-blended for 3 min at 300 rpm by the wet-mixing granulator. In order to get suitable wet mass, polyethylene glycol 400 and polyoxyl 35 castor oil were added to HPMC E5 (10% w/w) aqueous solution. And the resulting aqueous solution was then slowly added to the mixture of amoxicillin and Avicel PH 101 or PH 301 as mentioned above. The wet mass was extruded with a friction plate by the extruder. The extruded material was spheronized at 1500 rpm for 60 s by the spheronization to form pellet cores with appropriate diameter. Finally, the wet pellets were dried at 45°C for 3 h with a modified method [16]. The pellets were prepared by 10% HPMC (HPMC E5, 2% based on the cores w/w) aqueous solution using a fluid-bed coater named miniGlatt (Glatt, Germany). The conditions were given as follows: the temperature was 35 ~ 40°C, the spray rate was 5 ~ 7 ml/min, and the atomization pressure was 1.1 bar. The pellets coated with HPMC were saved in sealed pockets and closed containers.

#### Pulse Ⅱ and pulse Ⅲ

The obtained pellet core underwent further craft to produce pulse II and III pellets. The pulse Ⅱ was prepared by coating Eudragit L30 D-55. The coating suspension contained TEC (equaling to 100% of the dry polymer of Eudragit L30 D-55, w/w) as plasticizer, talc as anti-plastering aid agents. The procedure of coating was as follows. The dispersions were diluted with distilled water to achieve 20% content of dry polymer. And other materials were added slowly to the coating fluid with 500 rpm of stirring for at least 1 h. The pellets coated with HPMC were then coated with different coating weight of Eudragit L30 D-55 polymer (respectively 10%, 20%, 30% based on weight HPMC pellets, w/w) by a fluid-bed coater [17]. The conditions were given as follows: the temperature was 25 ~ 35°C, the spray rate was 3 ~ 5 mL/min, and the atomization pressure was 1.0 bar. After being taken out of the fluid-bed coater, pellets were rolled out on salvers, then kept in the oven for 8h at 50°C, finally saved in sealed pockets and closed containers.

The pulse Ⅲ was prepared by AQOAT AS-HF coating. The coating suspension contains AQOAT AS-HF, sodium lauryl sulfate (equaling to 0.5% of the dry polymer of AQOAT AS-HF, w/w) being the polymer to increase solubility of AQOAT AS-HF, TEC (equaling to 100% of the dry polymer of AQOAT AS-HF, w/w) as plasticizer and talc as anti-plastering aid agents. The procedure of coating was as follows. Sodium lauryl sulfate and TEC were dissolved in the water. AQOAT AS-HF was slowly added to the solution to achieve a dry polymer content of 7%. Talc was slowly added to the solution and stirred at 500 rpm for more than 2 h. The pellets coated with HPMC were then coated with different coating weight of AQOAT AS-HF polymer (respectively 5%, 15%, 25%, based on weight HPMC pellets, w/w) by a fluid-bed coater [17]. The conditions were given as follows: the temperature was 25 ~ 30°C, the spray rate was 4 ~ 6 mL/min, and the atomization pressure was 1.0 bar. The coated pellets were dried in a fluid-bed at atomizing pressure of 0.6 bar and entering temperature of 60°C for 30 min. Pellets were saved in sealed pockets and closed containers.

Box–Behnken design was used to optimize the tablet formulation. According to the results of the single-factor experiments, coating weight of Eudragit L30 D-55 (X_1_), coating weight of AQOAT AS-HF (X_2_), the extrusion screen aperture (X_3_) and compression forces (X_4_) were chosen as the main factors. The independent variables studied (X_1_, X_2_, X_3_ and X_4_) and the responses in the experimental design were shown in Table 1. The chosen dependent responses (R_1_, R_2_) were the cumulative release in the *in vitro* dissolution test with a rotating speed of 75 rpm. The experimental and the results for the 29 formulations were summarized in Table 2.

**Table 1:** Factors and response investigated in the experimental design.

Factors	Levels		
	-1	0	1
X_1_: coating weight of Eudragit L30 D-55 (%)	10	20	30
X_2_: coating weight of AQOAT AS-HF (%)	5	15	25
X_3_: extrusion screen aperture (mm)	0.3	0.4	0.6
X_4_: compression forces (KN)	8	13	18
Response	Constraints		
R_1_: cumulative release at 2.25h (%)	49.5 ~ 57.5		
R_2_: cumulative release at 4.5h (%)	79.0 ~ 85.0		

**Table 2:** Observed responses of Box–Behnken experimental design.

NO.	X_1_	X_2_	X_3_	X_4_	R_1_	R_2_
1	30	15	0.3	13	40.6	74.6
2	20	5	0.6	13	43.8	64.8
3	30	15	0.4	8	43.6	79.8
4	20	15	0.4	13	54.1	83.9
5	20	25	0.3	13	53.4	70.5
6	20	15	0.4	13	54.1	83.9
7	10	15	0.6	13	42.8	73.6
8	10	15	0.4	18	60.9	87.8
9	20	15	0.4	13	53.9	83.6
10	30	25	0.4	13	41.8	65.9
11	20	25	0.6	13	43.6	58.9
12	20	15	0.4	13	54.3	84.2
13	30	15	0.6	13	39.2	60.9
14	10	5	0.4	13	61.2	95.7
15	10	15	0.4	8	63.9	90.7
16	20	15	0.3	18	60.2	89.8
17	20	5	0.4	8	55.9	90.7
18	20	5	0.3	13	52.8	91.2
19	30	5	0.4	13	40.5	67.9
20	30	15	0.4	18	42.9	74.3
21	20	15	0.3	8	56.8	83.2
22	20	15	0.4	13	54.3	83.7
23	10	25	0.4	13	65	76.3
24	20	15	0.6	8	39.5	69.3
25	10	15	0.3	13	63.5	83.9
26	20	25	0.4	8	53.2	72.3
27	20	5	0.4	18	60.2	90.7
28	20	15	0.6	18	45.3	72.9
29	20	25	0.4	18	64.1	74.3

#### Compression of coated pellets and film coating

The pulseⅠ, Ⅱ, Ⅲ and other excipients were compressed by different compression forces (8KN, 13KN, 18KN) using a single-punch tableting machine (Riva Mini Press MⅡ, Riva, Germany) with a punch of 22 mm diameter by Box–Behnken design in Table 2. Avicel MCC PH 102 and colloidal silicon dioxide was selected as the glidant in the tablet compression procedure. Then, pulse Ⅰ, pulse Ⅱ, pulse Ⅲ, PVPP, magnesium stearate, Avicel MCC PH 102 and colloidal silicon dioxide were mixed and pressed to make the tablets with suitable compression force.

The tablets were prepared by film-coating of Opadry^®^ (Colorcon). The coating suspension was prepared by adding Opadry^®^ powder to the purified water with 500 rpm stirring for at least 45 min. The amoxicillin containing tablets were coated using the Opadry^®^ suspension by a coating pan (BY300, Shanghai precision instruments Co., Ltd, Shanghai, China). The conditions were given as follows: the temperature was 35 ~ 40°C, the spray rate was 3 ~ 6 mL/min, the atomization pressure was 1.5 bar and weightening ratio of film coating was 3%. The film-coated tablets were saved in sealed pockets and closed containers.

#### *In vitro* dissolution study

The dissolution test was performed using USP apparatus II (Paddle) with 900 mL solution at 37 ± 0.5°C in 3 stages: 50 mM potassium phosphate monobasic buffer at pH 4.0 (0–2 h), pH 6.0 (2–4 h) and pH 6.8 (4 h and beyond), with a rotating speed of 75 rpm, at designated sampling time points: 0.25, 0.5, 1, 2, 2.25, 2.5, 3, 4, 4.25, 4.5, 5 and 6h [18]. At predetermined intervals, 5 mL of the medium was extracted and filtered through a nylon membrane filter (0.22 μm). After each extraction, 5 mL medium maintained at 37 ± 0.5°C was immediately added for the purpose of compensation. The concentrations of amoxicillin in the withdrawn dissolution medium were determined by a High-Pressure Liquid Chromatography with UV Detector (HPLC-UV) method. The octadecylsilane chemically bonded silica chromatographic column (250 × 4.6 mm, 5 μm) was used for the separation. The mobile phase contained a mixture of 50 mM potassium dihydrogen phosphate aqueous solution (adjusted to pH 5.0 by 2 mol/L potassium hydroxide solutions): acetonitrile in the volume ratios of 60:40. The detection wavelength was 254 nm. The flow rate was 1.0 mL·min^-1^.The column temperature was 40°C and the injection volume was 10 μL.

#### Field emission scanning electron microscopy

The surface morphology of coated pellets before and after the compression was investigated by field emission scanning electron microscope (FESEM). The samples were sputter coated with a thin layer of silver under Argon atmosphere, and scanned by the FESEM (Zeiss Sigma, Germany).

### Pharmacokinetic study

#### Animals

Six male adult beagle dogs had an age range of 6 ~ 10 months and a weight range of 9.0 ~ 11.0 kg. The principles of Laboratory Animal Care were approved by the department of laboratory animal research at China State Institute of Pharmaceutical Industry (License Number: SYXK (Shanghai) 2014–0018). The use of animals was approved by the Animal Management and Ethics Committee of China State Institute of Pharmaceutical Industry. Prior to the test, all the dogs were fed chow and sterilized tap water in a standard laboratory.

#### Pharmacokinetic study

The multi-pulsatile release tablets and common tablets (250 mg, Kunming Yuanrui Pharmaceutical Co., Ltd., China) were used in the pharmacokinetic and the bioequivalence studies. A single multi-pulsatile release tablet (775 mg) or three common tablets (750 mg) was administered orally after a meal by the design of a randomized crossover study. The foreleg venous blood samples were collected into vacuum tubes immediately before and after the drug administration at the following time points: 0.5, 1, 1.5, 2, 2.5, 3, 4, 4.5, 5, 6, 8, 10 and 12 h. The blood samples were processed by centrifugation at 3600 rpm and at 4°C for 10 min. The obtained plasma samples were stored at −80°C until analysis.

#### Sample preparation

The plasma was thawed at room temperature. To all the standards (25 μL) or QC samples (25 μL), 50 μL of ampicillin trihydrate (IS) and 225 μL of blank plasma were added, and vortexed for 30 s. Then, methanol (0.7 mL) was added to the plasma samples, being subsequently vortexed for 30 s. The mixture was centrifuged at 12000 rpm at 4°C for 3 min. Then the supernatant was transferred into autosamper vials and subject to the Liquid chromatography–mass spectrometry / mass spectrometry (LC-MS/MS) analysis.

#### Bioanalytical method

The HPLC system consisted of a model CBM-20A system controller, a model LC-20AD pump, a model SIL-20A_HT_ auto-injector, a model CTO-20A column oven, and a model C-R7A plus integrator (Shimadzu, Kyoto, Japan). A Triple Quadrupole Mass Spectrometer (LCMS-8030, applied Shimadzu, Kyoto, Japan) was used for the LC-MS-MS analyses and detection. The MS was operated in positive ion detection mode [19]. Nitrogen was used as the nebulizing turbo spray. An octadecylsilane chemically bonded silica chromatographic column (50 × 4.6 mm, 3.5 μm) was used for the separation. The mobile phase was prepared by adding 2 mM glacial acetic acid aqueous solution to methanol (20:80, v/v). The flow rate was 1.0 mL·min^-1^ with a splitting ratio of 1/3 and the column temperature was 40°C. The injection volume was 10 μL.

The temperature of the vaporizer was set at 400°C. The drying gas (N_2_) flow rate was 15 L/min. The nebulizer gas (N_2_) flow rate was 3 L/min. The mass spectrometer was operated at unit mass resolution with a dwell time of 100 ms per transition. Collision energy for amoxicillin was set at -11 V and -20 V for IS. Quantification was performed using multiple reactions monitoring (MRM) of the transition ions m/z 366.00 → m/z 349.10 and m/z 350.00 → m/z 106.10 for amoxicillin and IS respectively.

### Pharmacokinetic data analysis

A concentration-time curve was plotted. The AUC_0-12 h_ and AUC_0-∞_ was calculated by the trapezoidal rule [20]. T_max_ (the time to reach the maximum concentration) and C_max_ (the maximum concentration), was directly acquired from the concentration time curve. All the pharmacokinetic parameters were calculated by the DAS Software (Version 2.0).

### Establishment of PBPK model

The Simcyp^®^ software (Simcyp Simulator, Version 13.2, Certara USA, Inc., USA) was used to establish the PBPK model of amoxicillin for human and beagle dog based on the physical and chemical properties of amoxicillin and the pharmacokinetics parameters reported in literature as summarized in Table 3. The Advanced Dissolution, Absorption and Metabolism (ADAM) model built in Simcyp^®^ was used to simulate the absorption process of amoxicillin formulations. ADAM model consists of eight compartments based on the anatomical structure of the small intestine, namely, duodenum, two segments of jejunum, four segments of ilium, and colon. Rodgers and Rowland method was used to predict the tissue-partition coefficients (P_t:p_) since amoxicillin is an ampholyte.

**Table 3:** Physical, chemical parameters and *in vitro* metabolic parameters amoxicillin used in PBPK model building.

Name	Input value	Source
Mol Weight (g·mol^-1^)	365.400	Literature [21]
Compound Type	Ampholyte	Literature [22]
Solubility (mg·mL^-1^) ^a^	3.4	Literature [21]
pKa_1_ ^a^	2.800	Literature [21]
pKa_2_ ^a^	7.200	Literature [21]
log P_o:w_ ^a^	-0.580	Literature [21]
P_eff_,man (10^−4^ cm·s^-1^) ^b^	1.47	Literature [22]
B/P ^c^	0.67	Predicted by Simcyp
fu value ^a^	0.63	Literature [23]
CL_po_(L·h^-1^·Kg^-1^)^a^	0.045	Literature [21]

a, Measured values

b, Optimized values

c, Predicted values

log P_o:w_, the oil-water partition coefficients; P_eff_,man, Human jejunum permeability; B/P, Blood/plasma ratio; fu value, Fraction unbound in plasma; CL_po_, oral clearance.

#### Data statistical analysis

The Box–Behnken design statistical analysis Box–Behnken design was performed using Software Design-Expert (Design-Expert 8.0.6, Stat-Ease, Inc., USA) and Origin Pro Software (Origin Pro 8.5.1, Origin Lab Co., Ltd., USA). The pharmacokinetic studies statistical analysis data and results were performed by a two one-sided t-test, and a P value < 0.05 was accepted as significant by DAS (DAS Version 2.0, Shanghai, China).

## Results and Discussion

### Preparation of the three-pulse release tablets and its optimization

#### PulseⅠ

The excipient Avicel MCC PH 101 or PH 301 was chosen due to their capacity to enhance the buffering capacity of pulse Ⅰ, and that is crucial to the tablet compression procedure. The concentration of the binder HPMC E 5 solution was fixed at 5%, 10% and 15% considering the particle size distribution of granules. The composition of different formulation of the pulseⅠwe examined was shown in Table 4. It was found the uniformity of mixing was mainly determined by the particle size distribution (PSD) and the angle of repose. More than 70% of the PSD should be in 105 ~ 500 μm and the angle of repose should be below 35° according to the literature [24, 25].

**Table 4:** The composition and evaluation index of pulseⅠ formulation.

formulation	F1	F2	F3	F4	F5	F6
Amoxicillin(%)	69	69	69	69	69	69
Avicel PH 301(%)	25	25	25	/	/	/
Avicel PH 101(%)	/	/	/	25	25	25
HPMC E5(%)	4 (5%*)	4 (10%*)	4 (15%*)	4 (5%*)	4 (10%*)	4 (15%*)
PVPP(%)	2	2	2	2	2	2
500μm >PSD>105μm(%)	64.2	80.1	67.1	59.2	70.7	66.5
angle of repose (°)	36.3	27.8	34.6	41.4	35.2	35.6

F1 ~ F6, is formula 1 ~ formula 6 respectively; PSD, particle size distribution.

* the solution concentration of HPMC E5.

It can be seen from the Table 4 that the excipient Avicel MCC PH 301 was much better than PH 101 as for the formation of particles under the same conditions. It might be due to the fact that the fiber of PH 301 is longer than that of PH 101. Long PH 301 fiber is easier to protect the integrated pellets, and avoid their being destroyed in the compression process of tablet. Our experimental results also showed that PH 301 could prevent the pellets from being damaged. This is consistent with the experimental results by Xiaole Qi *et al* [26]. The best formulation of pulseⅠwas finally determined to be amoxicillin 69%, Avicel PH 301 25%, HPMC E5 4% and PVPP 2%.

#### Pulse Ⅱ and pulse Ⅲ

It is crucial to maintain the humidity to some extent during the process of the extrusion. This is also important to get the uniformity of the pellets [27–29]. Good extrusion requires the lubrication of the materials by non-ionic surface active agent, which can in the same time prevent water in the material from evaporating too fast in the process of extrusion [15, 30]. Drug loading is quite high in the present study. Therefore it is necessary to add non-ionic surface active agent in the binder solution. We chose water-soluble PEG 400 and castor oil 35 to guarantee the smoothness and completeness of pellets in the extrusion/spheronization process.

**Fig 3:**
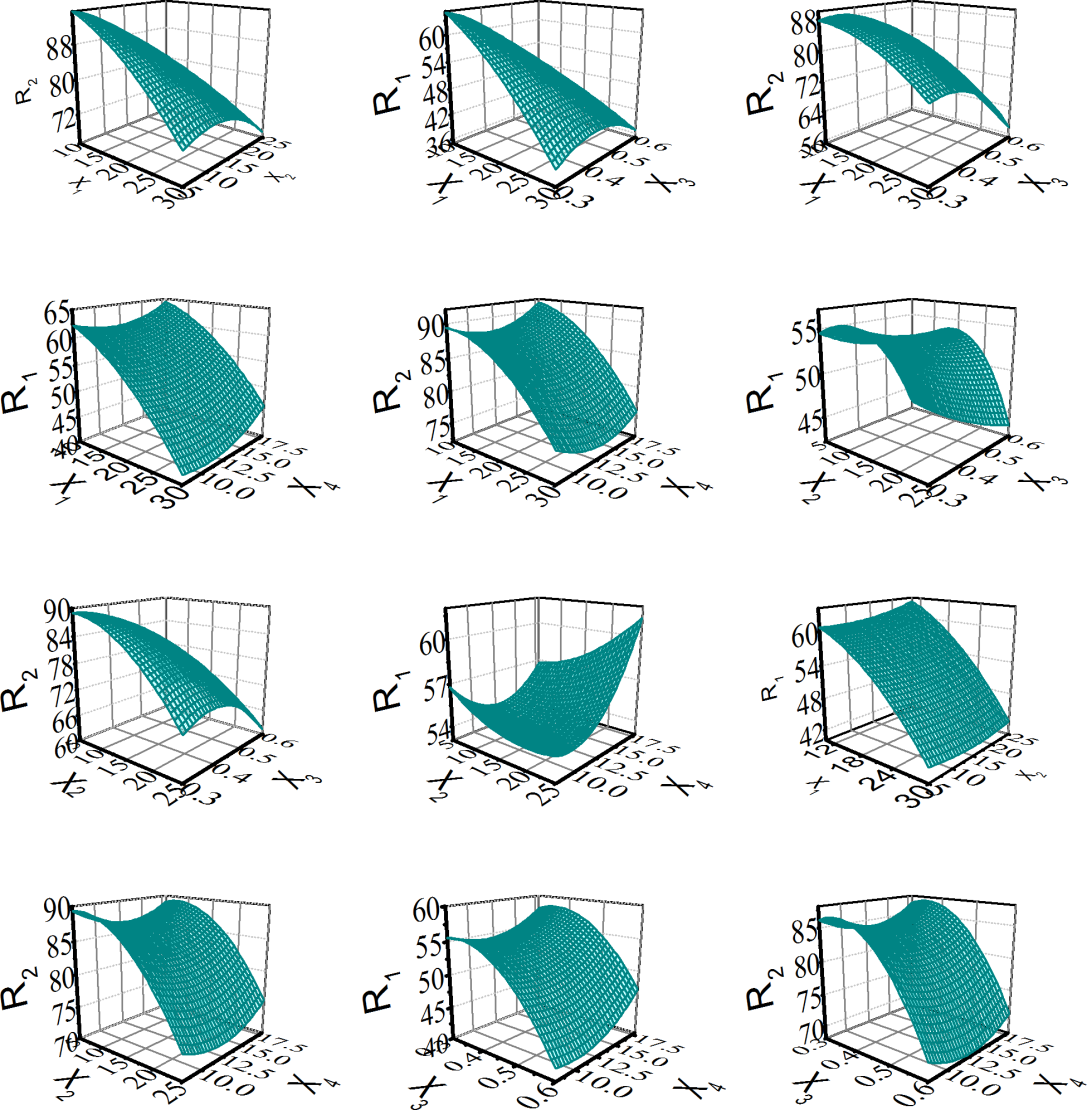
Effect of variables (X_1_, X_2_, X_3_, X_4_) on the response R_1_ and R_2_: the response surface plots. X_1_: coating weight of Eudragit L30 D-55; X_2_: coating weight of AQOAT AS-HF; X_3_: the extrusion screen aperture; X_4_: compression forces; R_1_ and R_2_: the cumulative release at 2.25h and 4.5h.

Box–Behnken designs [31] are experimental designs aiming to investigate the response surface methodology, and each design can be thought of as a combination of a two-level factorial design with an incomplete block design. Based on the single factor experiment, four factors and three levels Box–Behnken design was carried through to get an optimal composition of the tablets by investigating the effect of the coating weight of Eudragit L30 D-55, the coating weight of AQOAT AS-HF, the extrusion screen aperture and the compression forces. The dependent variables and independent variables were related by mathematical relationships [26]. The polynomial equations obtained were:

R_1_ = +92.76396–1.31586*x_1_-0.43943*x_2_+49.93199*x_3_-3.19882*x_4_-6.25000E-003*x_1_*x_2_+3.49828*x_1_*x_3_+0.01150*x_1_*x_4_-0.21897*x_2_*x_3_+0.033000*x_2_*x_4_+0.98276*x_3_*x_4_-0.027583*x_1_^2+9.41667E-003*x_2_^2–188.88889*x_3_^2+0.092667*x_4_^2 (R^2^ = 0.9361); R_2_ = +95.69340+0.10771*x_1_-1.30345*x_2_+150.19789*x_3_-2.44979*x_4_+0.043500*x_1_*x_2_-0.20690*x_1_*x_3_-0.013000*x_1_*x_4_+2.34828*x_2_*x_3_+1.00000E-002*x_2_*x_4_-6.89655E-003*x_3_*x_4_-0.030250*x_1_^2–0.045500*x_2_^2–258.61111*x_3_^2+0.10100*x_4_^2 (R ^2^ = 0.9361);

The interaction among the independent variables (X_1_, X_2_, X_3_ and X_4_) in the equations and the interaction between the independent variables and the dependent variables (R_1_, R_2_). The coefficients before (X_1_ ~ X_4_) indicated the independent variables were dependent on each other. The coefficients with more than one factor term and those with higher order terms represented interaction terms and quadratic relationships [26]. A plus sign expresses a synergistic effect and a subtraction sign indicates an antagonistic effect [26]. The independent values of X_1_ ~ X_4_ were ascertained to obtain the prediction values of R_1_ and R_2_ in the equation. The relation between the independent variables and dependent variables were further illuminated by the contour plots in Fig 3.

**Fig 4:**
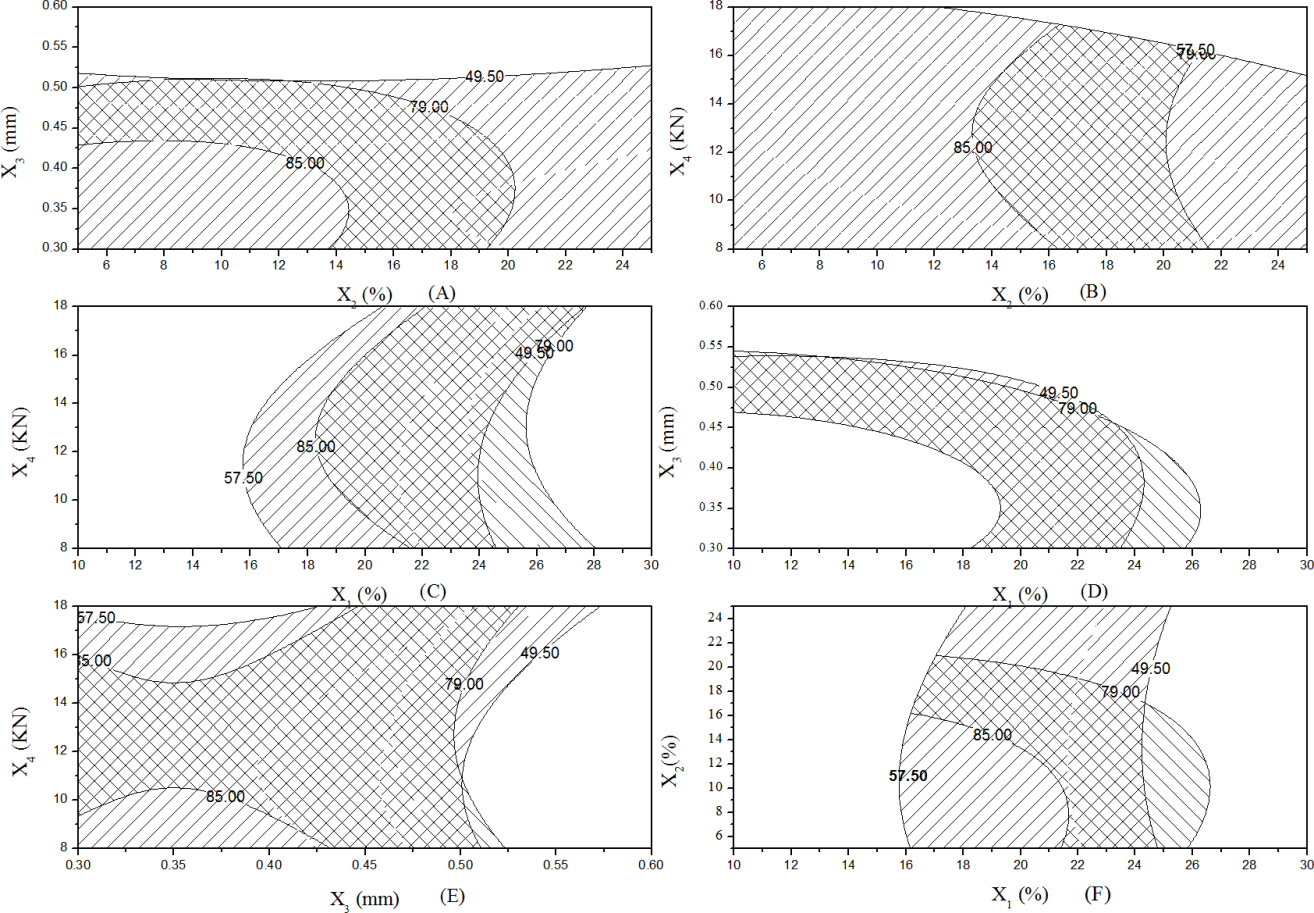
Effects of Variables (X_1_, X_2_, X_3_, X_4_) on Y_1_ and Y_2_: the overlay contour plots. A, effect of X_2_, X_3_ variables on the response R_1_ and R_2;_ B, effect of X_2_, X_4_ variables on the response R_1_ and R_2;_ C, effect of X_1_, X_4_ variables on the response R_1_ and R_2;_ D, effect of X_1_, X_3_ variables on the response R_1_ and R_2;_ E, effect of X_3_, X_4_ variables on the response R_1_ and R_2;_ F, effect of X_1_, X_2_ variables on the response R_1_ and R_2._

When the extrusion screen aperture (X_3_) and the compression forces (X_4_) were fixed values, R_1_ or R_2_ were increasing as the coating weight of Eudragit L30 D-55 (X_1_) and the coating weight of AQOAT AS-HF (X_2_) were decreasing in the Fig 3 and the Fig 4 (F). As seen from the Fig 3 and the Fig 4A, 4D and 4E, when the coating weight of Eudragit L30 D-55 (X_1_), the coating weight of AQOAT AS-HF (X_2_) and the compression forces (X_4_) were fixed values, R_1_ or R_2_ were increasing as the extrusion screen aperture (X_3_) were decreasing. It was illuminated that the extrusion screen aperture had significant impact on the release of pellets in the preparation craft. When the weight of Eudragit L30 D-55 (X_1_), the coating weight of AQOAT AS-HF (X_2_) and the extrusion screen aperture (X_3_) were fixed values, R_1_ and R _2_ were increasing as the compression forces (X_4_) were increasing in the Fig 3 and the Fig 4B, 4C and 4E. It was explained that the coated membrane was damaged beyond a critical pressure. When the coating weight of AQOAT AS-HF (X_2_), the extrusion screen aperture (X_3_) and the compression forces (X_4_) were fixed values, R_1_ was outside of the R_1_ range (R_1_, 49.5 ~ 57.5%) and R_2_ was inside of the R_2_ range (R_2_, 79.0 ~ 85.0%), as the coating weight of Eudragit L30 D-55 (X_1_) was decreasing in the Fig 3 and the Fig 4C, 4D and 4F. It was illuminated that the coating weight of Eudragit L30 D-55 (X_1_) impact was significant R_1_ than R_2_. When the weight of Eudragit L30 D-55(X_1_), the extrusion screen aperture (X_3_) and the compression forces (X_4_) were fixed values, R_1_ was inside of the range (R_1_, 49.5 ~ 57.5%) and R_2_ was outside of the range (R_2_, 79.0 ~ 85.0%), as the coating weight of AQOAT AS-HF (X_2_) was increasing in the Fig 3 and the Fig 4A, 4B and 4D. It was illuminated that coating weight of AQOAT AS-HF (X_2_) impact was significant R_2_ than R_1_. We found that the predicted values were compared with the measured values in reasonably close agreement in Table 5. The model predicted R_1_ and R_2_ of 54.4 and 83.9 at the coating weight of Eudragit L30 D-55 (X_1_), the coating weight of AQOAT AS-HF (X_2_), the extrusion screen aperture (X_3_) and the compression forces (X_4_) values of 20, 15, 0.3 and 13 respectively.

**Table 5:** Measured values and predicted values of the response of Box-Behnken design.

Evaluating indexes	Observed	Predicted
R_1_	51.7	54.4
R_2_	83.9	83.9

#### The influence of compression on coating of the pellets

Different compression forces influenced coating the film integrity during the compression progress. The ideal coating should be stable and strong enough and keep its physical continuity to have a large extension before being destroyed [32]. During the compression progress, it is impossible to avoid the damage and transformation of the coat, which plays a significant role in maintaining the release of pellets. It has also been reported that stretching of an intact polymer layer is advantageous to the film permeability [33]. And the compression was leaded to the destroyed of film to increase the drug release [34]. Another reason might be the deformation pressure of the pellets, which in turn can increase the surface area of the pellets, thus increasing the drug release rate [32]. The effect of pressing force on the drug release was investigated using Box–Behnken design as shown in Fig 3. Varying compression force was found to affect the drug release and the integrity of coating film. Finally, it was demonstrated that the pressed force of 13 KN could ensure the integrity of coating film.

#### *In vitro* dissolution and FESEM

**Fig 5:**
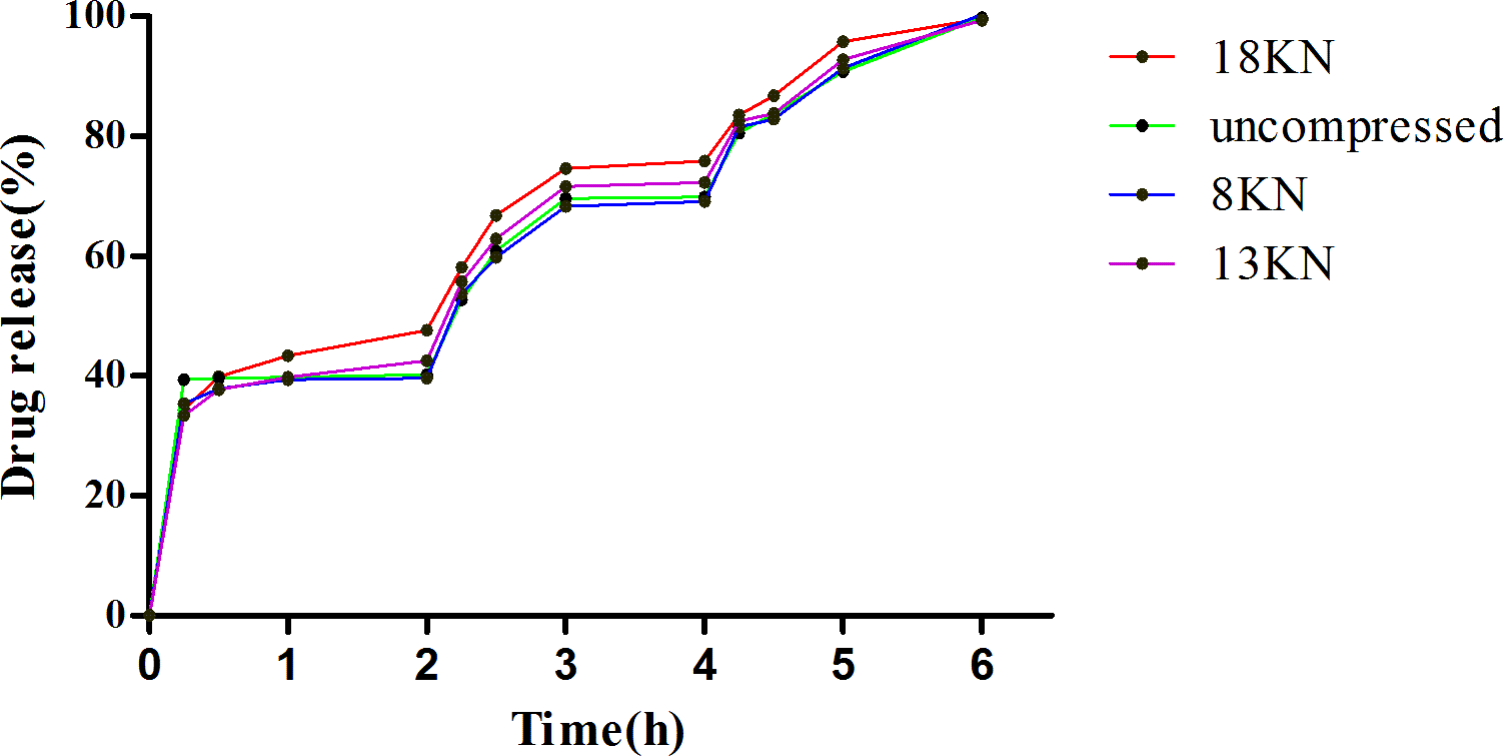
Drug release for pulse tablets of amoxicillin with uncompressed and under compression forces of 8 KN, 13 KN and 18 KN, respectively (n = 6).

After preliminary investigation, a speed of 75 rpm was chosen to investigate the dissolution for the comparison of uncompression and different pression force (Fig 5). It has been shown that pH dependent coating began to dissolve in suitable pH and the pulsatile tablets of amoxicillin presented three pulses release profiles in *in vitro* dissolution.

**Fig 6:**
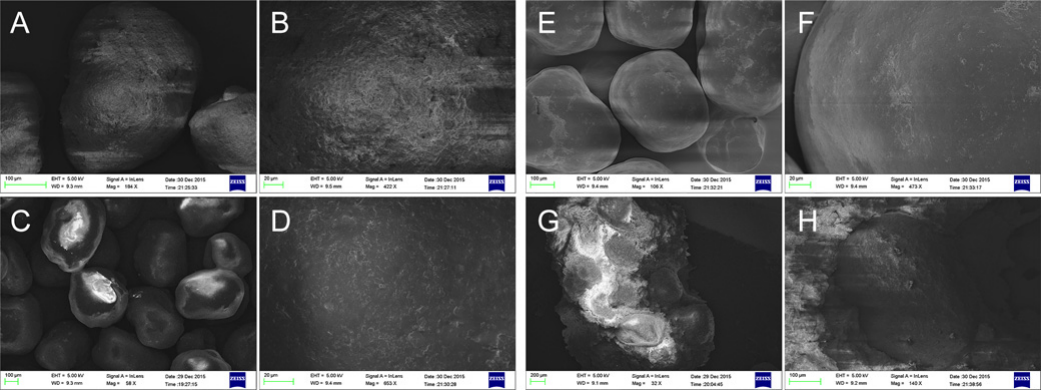
Scanning electron micrographs of pellets: (A), (B) uncoated pellets; (C), (D) L30D-55 coated pellets; (E), (F) AS-HF coated pellets; (G), (H) after pressed with 50% pellets under compression force of 13 KN.

FESEM photos are shown in Fig 6, when uncoated pellets, L30 D-55 coated pellets, AS-HF coated pellets and the pulsatile tablets made under the compression force of 13 KN. FESEM photos showed that the compression of pellets into the tablets didn’t damage the form of pellets as for the surface. Hence, it was determined the optimum preparation of the three-pulse release tablets of amoxicillin and the pression force could at least maintain the integrity of the film surface.

### Pharmacokinetics

Linearity of the calibration curve was yielded in the concentration range from 105.60 to 83633.90 ng·mL^-1^ (r^2^ ≥ 0.996). The intra-day and inter-day precision (n = 6) was all less than 5.0%. The mean recoveries of amoxicillin at QC concentrations (316.80, 34847.46 and 66907.11ng·mL^-1^) were 91.7 ± 2.7%, 93.4 ± 3.9% and 92.6 ± 3.2% (n = 3), respectively. In storage for 3 days at −80°C and three freeze–thaw cycles, amoxicillin remained stable in plasma. And, the results manifested that the bioanalytical method was qualified to quantify the plasma concentration for the pharmacokinetic studies.

**Fig 7:**
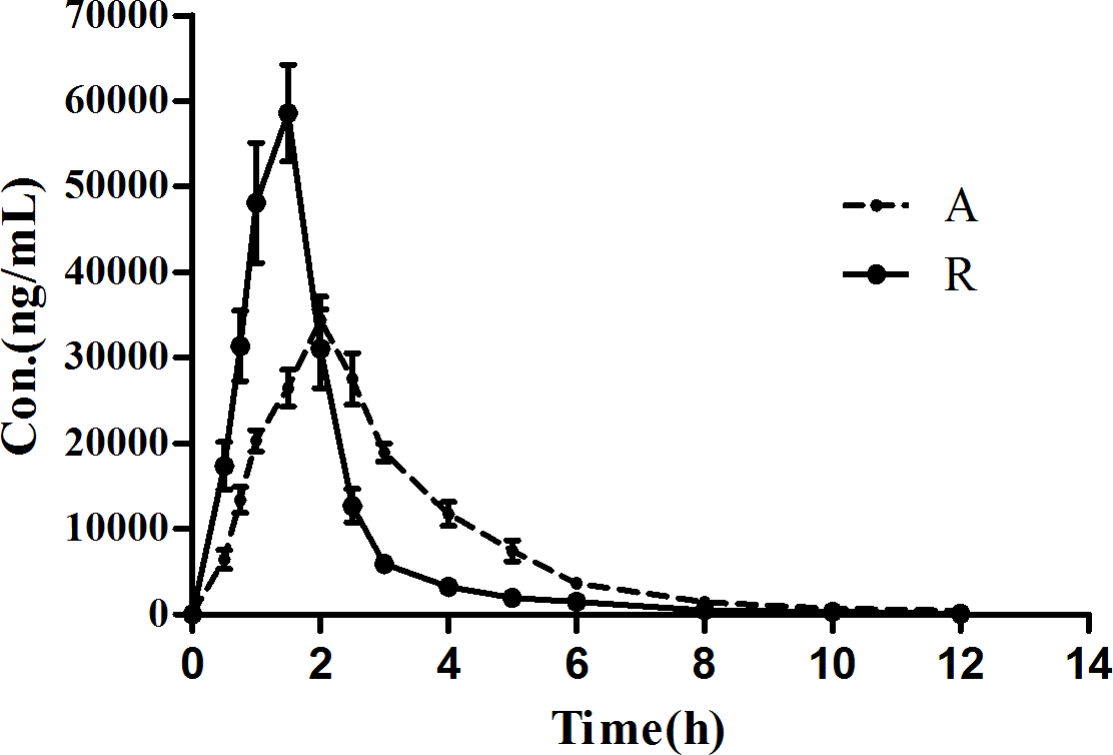
Plasma concentration time curves of amoxicillin in beagle dogs (n = 6). A: amoxicillin pulse tablets; R: amoxicillin tablets

Extrapolation method for AUC_0-∞_ was estimated by a linear regression model from the last three data points, which has been log-transformed, in the elimination phase. C_max_ was received from the actual surveyed concentration without interpolation. Plasma concentration time profiles of amoxicillin in the beagle dogs given pulsatile release tablets and reference tablets are shown in Fig 7. A study has shown that under the fasting conditions the amoxicillin AUC was significantly lower than that after the intake of the breakfast. This might be due to the early gastric emptying, and also the poor absorption from the deeper parts of the small intestine. Therefore, the amoxicillin tablets are usually administered orally after a meal [35]. The pharmacokinetic parameters and data of bioequivalence studies are tabulated in Table 6. The significant difference between the two formulations was also confirmed by the statistical analysis of two one-sided t-test. Compared to the formulation of the amoxicillin oral tablet, the pharmacokinetic profiling of the pulsatile tablet demonstrated its characteristics of the sustained release in beagle dogs.

**Table 6:** Pharmacokinetics parameters in beagle dogs after oral administration, n = 6, mean±S.D.

Pharmacokinetic parameters	Unit	Amoxicillin pulse tablets	Amoxicillin tablets(common tablets)
AUC_0-12 h_ /Dose	×10^−6^ mL^-1^·h	141.17 ± 25.41	133.38 ± 25.14
AUC_0-∞_ /Dose	×10^−6^ mL^-1^·h	141.17 ± 25.41	133.38 ± 25.14
T_max_	h	2.08 ±0.49	1.25 ± 0.27
C_max_ /Dose	×10^−6^ mL^-1^	46.76 ± 6.75	75.56 ± 8.34
t_1/2_	h	1.67 ±0.27	1.30 ± 0.38
MRT_0→12 h_	h	3.01 ±0.19	2.05 ± 0.28

AUC_0-12 h_, area under the concentration time curve from zero to 12 h; AUC_0-∞_, area under the concentration time curve from zero hour to infinity; T_max_, time point of maximum plasma concentration; C_max_, maximum plasma concentration; t_1/2_, elimination half-life; MRT_0→12 h,_ the average retention time.

### PBPK model

**Fig 8:**
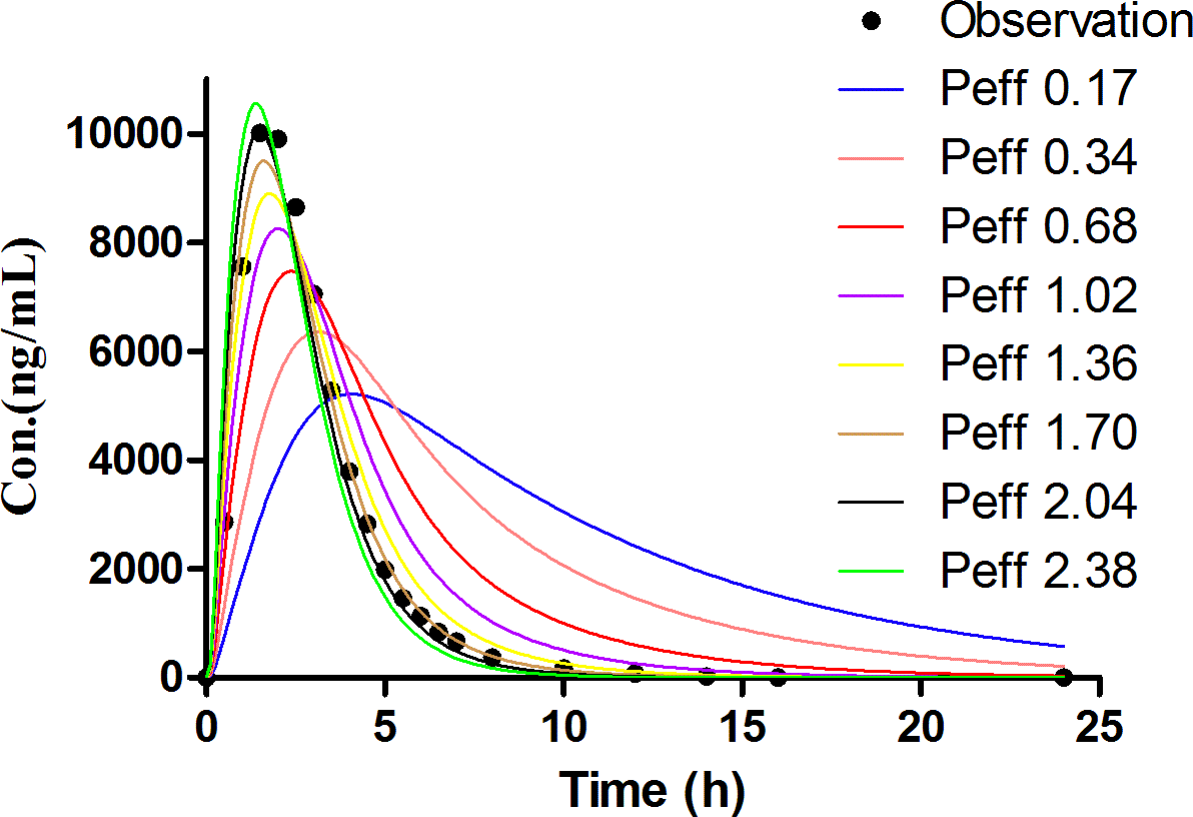
The parameter sensitivity analysis for the effect of P_eff_,man on the pharmacokinetic profile of amoxicillin suspension (750 mg).

**Fig 9:**
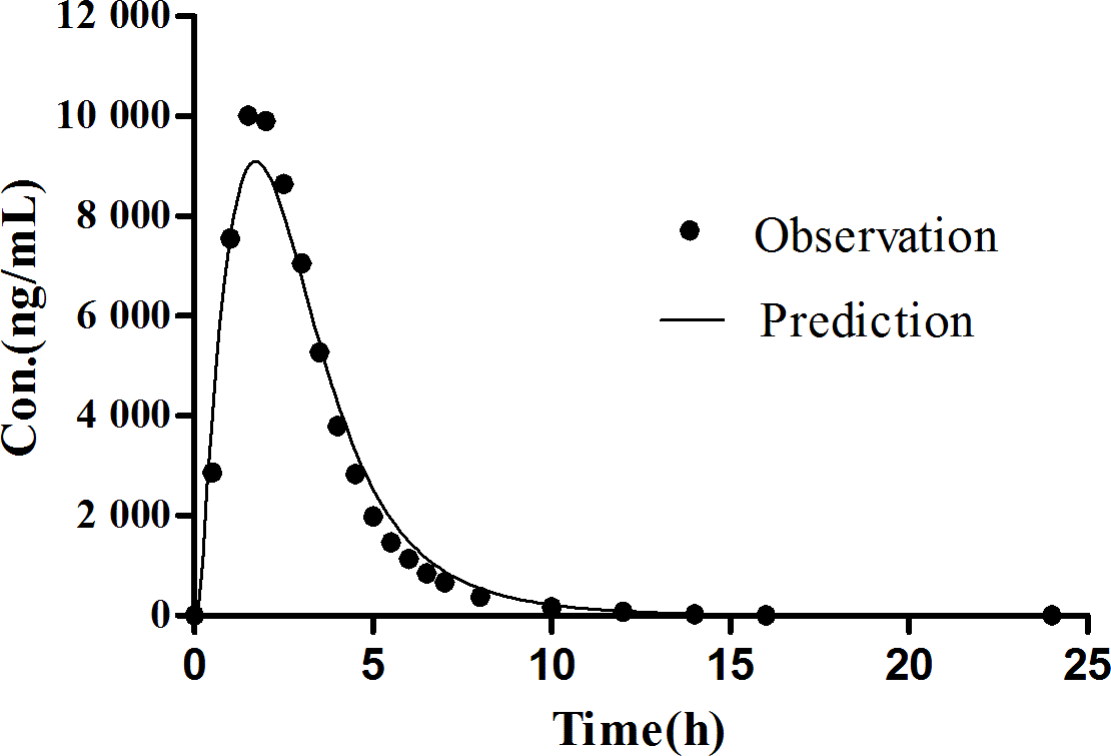
Predicted and observed plasma concentration time curve of amoxicillin suspension (750 mg) in human.

**Fig 10:**
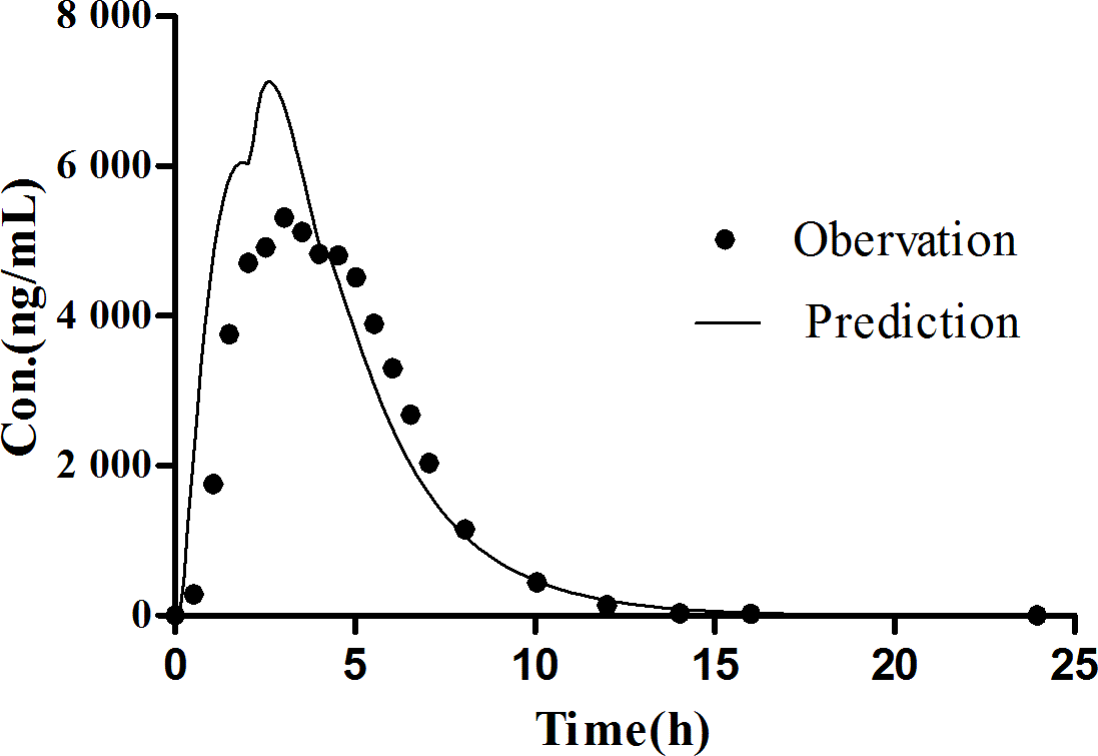
Predicted and observed plasma concentration time curve of amoxicillin pulse release tablet (775mg) in human.

**Fig 11:**
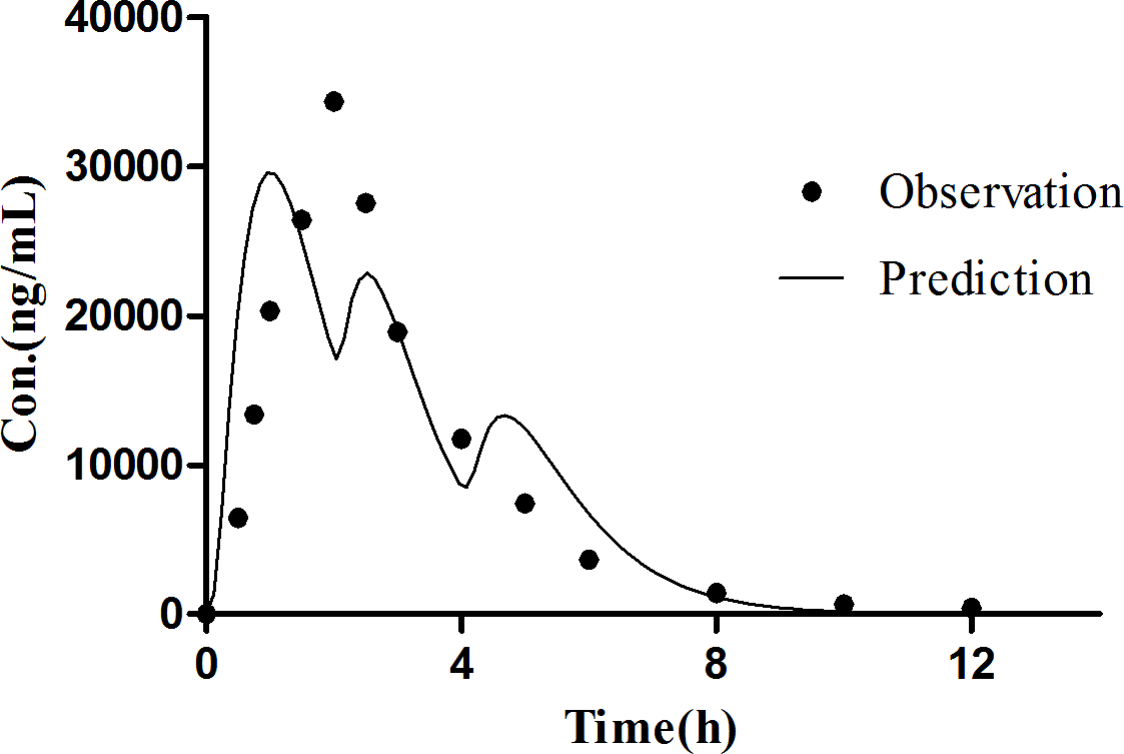
Predicted and observed plasma concentration time curve of amoxicillin pulse release tablet (775 mg) in beagle dog.

The Simcyp^®^ software is a platform and database for the ‘bottom-up’ mechanistic modelling and simulation dealing with the processes of oral absorption, tissue distribution, metabolism and excretion of drugs and drug candidates in healthy and disease populations [36, 37]. The computer simulation technology has successfully established *in vivo* absorption curve model and evaluated the bioequivalence of the amoxicillin capsule [38]. This study was to employ the Simcyp^®^ software to establish the *in vivo* absorption curve model of amoxicillin pulsatile tablet in both human and beagle dogs. PBPK model of amoxicillin was firstly developed for human. During the process of constructing model, the P_eff,_ man value of amoxicillin was obtained from a study which used Loc-I-Gut^®^ perfusion tube [22]. The initial value of P_eff,_ man was set at the mean value from 14 health volunteers, namely, 0.37×10^−4^ cm·s^-1^. However, the predicted C_max_ was always underestimated, resulting in an unsatisfactory fitting of observation data. Therefore, parameter sensitivity analysis (PSA) was conducted to estimate the influence of the input parameters in the PBPK model of human. PSA demonstrated that increasing P_eff_,man helped a lot in the prediction of pharmacokinetic profiles as shown in Fig 8. Then the range of the individual P_eff,_ man values from the literature were examined, and P_eff,_ man of amoxicillin was optimized to 1.47×10^−4^ cm·s^-1^, equaling to the highest P_eff,_ man value among the 14 subjects. Finally, the prediction was nicely fitted to the observed pharmacokinetic profiles of amoxicillin suspension in healthy volunteers [39] at a dose of 750 mg as shown in Fig 9 and Table 7. After the predictive capacity of amoxicillin PBPK model was confirmed, the *in vitro* dissolution release profile (Fig 5) of our in-house pulsatile amoxicillin tablet was input in the PBPK model to predict the pharmacokinetic behavior of the pulsatile tablet. As shown in the Fig 10 and Table 8, the predicted pharmacokinetic profile is quite close to the observed pharmacokinetic profile of commercial MOXATG^®^. Although being not so perfect as the prediction of amoxicillin suspension, the predictive performance for the pulsatile formulation is acceptable considering the fact that several mechanism has not been in the PBPK model such as the movement pattern of different pulse pellets along the gastrointestinal tract. Amoxicillin is usually referred to as a BCS III drug with high solubility but low permeability. There have been some cases of using PBPK to predict the pharmacokinetic profile of certain formulations of a BCS III drug recently, such as MK-0941, a drug development candidate [40]. Therefore, our study supports the potential utility of PBPK model as a useful tool to guide the formulation development for BCS III drug. To further confirm the reliability of PBPK model, a PBPK model of dog for amoxicillin was also built to predict the pharmacokinetic profile of our in-house pulsatile amoxicillin tablet. Amoxicillin-specific parameters were assumed to be same for human and dog. The prediction data was compared with our observation data from beagle dogs. A good fitting was also observed as shown in Fig 11 and Table 8. Interestingly, three pulses of amoxicillin release could be seen in the predicted plasma concentration-time curve, but not observed in the measured curve. One possible explanation for this discrepancy could be that the actual transit process of pulse pellets along the gastrointestinal tract might be shorter than that in the PBPK model. Thus, the interval between the triggering of different pulses in beagle dogs are shorter than that in the simulated scenario, leading to the disappearance of pulsatile release *in vivo*. Further study is warranted to investigate such hypothesis. The predication data of both human and dog indicates that the three pulse of amoxicillin release is superior to the tablet of immediate release and amoxicillin pulse release tablet has been improved significantly in the aspect of sustained releasing.

**Table 7:** The predicted and observed pharmacokinetic parameters of amoxicillin suspension (750 mg) in human.

	Predicted	Observed [39]	Fold error
C_max_ (μg·mL^-1^)	9.10	10.57	1.16
AUC_0-∞_ (μg·mL^-1^·h)	34.30	33.28	1.03
T_max_	1.68	1.75	1.04

**Table 8:** The predicted and observed pharmacokinetic parameters of pulsatile amoxicillin tablet (775 mg) in human and dogs.

	beagle dog			human		
	Predicted	Observed	Fold error	Predicted	Observed	Fold error
C_max_ (μg·mL^-1^)	29.64	35.07	1.18	7.14	6.62	1.08
AUC_0-∞_ (μg·mL^-1^·h)	105.09	105.87	1.01	34.09	29.79	1.14
T_max_	1.16	2.08	1.79	2.64	3.14	1.19

#### Conclusion

Experimental results manifested that an extrusion/spheronization and different pH sensitive coating materials preparation is successfully applied to a pulsatile release tablet to get pulsatile release in *in vitro* dissolution. A Box–Behnken design has been successfully used to clarify the interrelation of factors affecting the formulation, thus helping to optimize the formulation development. The sustained release of amoxicillin was actually achieved in beagle dogs. PBPK model of both human and dogs constructed with Simcyp^®^ could adequately describe the pharmacokinetic profile of pulsatile amoxicillin tablet. This study provides an example of using PBPK to guide the pulsatile formulation.
